# Improved Tool for Predicting Skin Irritation on Reconstructed Human Epidermis Models Based on Electrochemical Impedance Spectroscopy

**DOI:** 10.3390/bios13020162

**Published:** 2023-01-20

**Authors:** Manuel Chacón, Natalia Vázquez, Sergio Alonso-Alonso, Mairobi Persinal-Medina, Sara Llames, Marta Pevida, Ignacio Alcalde, Jesús Merayo-Lloves, Álvaro Meana

**Affiliations:** 1 Instituto Universitario Fernández-Vega, Fundación de Investigación Oftalmológica, Universidad de Oviedo, Avda. Doctores Fernández-Vega, 33012 Oviedo, Spain; 2 Instituto de Investigación Sanitaria del Principado de Asturias (ISPA), Avda. del Hospital Universitario, 33011 Oviedo, Spain; 3 Centro de Investigación Biomédica en Red en Enfermedades Raras (CIBERER) ISCIII, 28029 Madrid, Spain; 4 Unidad de Ingeniería Tisular, Centro Comunitario de Sangre y Tejidos de Asturias (CCST), 33006 Oviedo, Spain; 5 Instituto de Investigación Sanitaria-Fundación Jiménez Díaz (IIS-FJD), 28015 Madrid, Spain

**Keywords:** electrochemical impedance spectroscopy, capacitance, reconstructed human epidermis, skin irritation, safety assessment, alternative method

## Abstract

The rabbit skin irritation test has been the standard for evaluating the irritation potential of chemicals; however, alternative methods that do not use animal testing are actively encouraged. Reconstructed human epidermis (RhE) models mimic the biochemical and physiological properties of the human epidermis and can be used as an alternative method. On RhE methods, the metabolic activity of RhE models is used to predict skin irritation, with a reduction in metabolic activity indicating a reduced number of viable cells and linking cell death to skin irritation processes. However, new challenges have emerged as the use of RhE models increases, including the need for non-invasive and marker-free methodologies to assess cellular states. Electrochemical impedance spectroscopy (EIS) is one such methodology that can meet these requirements. In this study, our results showed that EIS can differentiate between irritant and non-irritant chemicals, with a significant increase in the capacitance values observed in the irritant samples. A ROC curve analysis showed that the prediction method based on EIS met OECD TG 439 requirements at all time points and had 95% within-laboratory reproducibility. Comparison with the MTT viability assay showed that prediction using EIS achieved higher sensitivity, specificity, and accuracy. These results suggest that EIS could potentially replace animal testing in the evaluation of irritation potential and could be a valuable addition to in vitro testing strategies.

## 1. Introduction

The rabbit skin irritation test has been the gold standard for the evaluation of the irritation potential of chemicals since the adoption of the OECD test guideline (TG) 404 in 1981 [1]. This method is based on the application of a single dose to the skin of an experimental animal and the scoring of the degree of irritation/corrosion at specified intervals. In the interest of both sound science and animal welfare, alternative methods to animal experimentation are highly encouraged to replace the testing and evaluation strategy provided in OECD TG 404. As so, OECD TG 439, originally adopted in 2010, includes in its latest update seven validated test methods of different commercially available in vitro models, representing an in vitro alternative based on the reconstructed human epidermis (RhE), a test system that mimics the biochemical and physiological properties of the native human epidermis [2]. These methods all comprise non-transformed human-derived epidermal keratinocytes, which have been cultured to form a multi-layered, highly differentiated model of the human epidermis [2]; however, RhE production differs on each test method since production is partly based on confidential and legally protected protocols. Therefore, the development and validation of novel RhE-based test methods are being actively encouraged through the guidance document No. 220, a document containing the performance standards to determine the reliability and relevance of similar skin irritation test methods that are structurally and mechanistically similar to the RhE test methods adopted in OECD TG 439 [3].

All RhE-based methods included in the OECD TG 439 for the identification of irritant compounds base their prediction on the study of cellular metabolic activity through the MTT viability assay after the application of the test products. A reduction in the metabolic activity of RhE models is thus associated with a reduced number of viable cells, linking cell death to skin irritation processes [2]. However, as the use of RhE models replaces animal experimentation, new challenges are expected to emerge that will need to be taken into account. Among them, the use of non-invasive and marker-free methodologies for the assessment of cellular states will be required as models are expected to increase in complexity, as with its integration in organ-on-a-chip systems [4,5]. In this sense, electrochemical impedance spectroscopy (EIS) emerges as a methodology capable of meeting these requirements.

In the last few years, EIS has established itself as one of the most popular analytical techniques in material research and is used in a variety of areas and analyses such as corrosion studies [6]; monitoring the ionic properties of polymers, colloids, and conductive coatings [7]; energy storage and battery analysis [8]; biological analysis and biomedical sensor development [9]; or studies of electrochemical kinetics, reactions, and processes [10]. EIS is therefore a very useful technique that can be used as a biological tool to evaluate the status and variations in RhE models after the application of different test compounds, having already shown to be useful in the assessment of eye irritation/severe eye damage in reconstructed human corneal epithelium models [11]. This is because RhE models are systems composed of conductive and dielectric elements. In an RhE model, the intracellular and extracellular medium behaves as an electrolyte, while the cell membranes form an electrical insulator [12]. EIS evaluation also allows differentiating between the effects caused by mostly capacitive elements (cell membranes) and mostly resistive elements (intercellular junctions) [13,14,15,16], resulting in the possibility to perform an independent analysis of the effect on RhE models, both at the cellular and structural level.

In short, this study presents the usefulness of EIS as a stand-alone tool to study skin irritation in QileX-RhE, an in-house RhE model [17]. Our results indicate that it is possible to identify irritant compounds in a non-invasive assay, representing an improvement in accuracy compared to current methods, which are based on cell viability, and paving the way for the implementation of EIS as a new tool to be used in regulated assays aimed at replacing animal experimentation.

## 2. Materials and Methods

### 2.1. Essential Test Method Components as Described in OECD TG 439

#### 2.1.1. General Conditions

Normal human epidermal keratinocytes were isolated from skin biopsies obtained from anonymous deceased organ donors after informed consent according to ethical approval granted by the Ethical Committee of Asturias (nº 2020.050) according to Spanish regulations for human, tissues, and tissue-based products. In brief, human keratinocytes were isolated, expanded, and cryopreserved according to previously described protocols [17]. Subsequently, human keratinocytes were thawed and expanded in a serum-free chemically defined media (CellNTec, Bern, Switzerland), seeded in 1.12 cm^2^, 0.4 µm pore size Transwell^®^ inserts (Corning, NY, USA) and cultured at the air-liquid interface for 14 days at 37 °C.

All cell strains were screened for bacteria, yeast, and fungi. Additionally, all skin donors tested negative for HIV, hepatitis B, and hepatitis C.

#### 2.1.2. Morphology

Random QileX-RhE samples were fixed in 10% formalin and embedded in paraffin. Histological sections were stained in hematoxylin–eosin and examined under light microscopy. The number of cell layers and the presence of a keratinized surface were evaluated according to recommendations.

#### 2.1.3. Barrier Function

Homogeneity of the integrity of the epithelial barrier effect in the QileX-RhE models was assessed by recording transepithelial electrical resistance (TEER). For this purpose, a Millicell-ERS2 multimeter (Merck, Rahway, NJ, USA) coupled to a pair of Ag/AgCl electrodes was used for TEER measurements. Cell models were brought to room temperature and the electrodes were immersed in such a way that one of them was on the inside of the insert and the other on the outside, keeping both stable at an angle of 90° with respect to the culture plate.

TEER values obtained in this study were compared to historical values and defined thresholds [17].

#### 2.1.4. Lipid Profile

The lipid content of the QileX-RhE model was analyzed in triplicate using a pool of two QileX-RhE models each time. Briefly, QileX-RhE models were frozen at −80 °C for 24 h and sent to the Lipid Analysis Service of the Universidad de la Laguna. The QileX-RhE models were mechanically separated from the inserts and the total lipids were extracted according to the methodology of Bligh and Dyer. The lipid extract was then dried in nitrogen atmosphere, weighted, and the lipid composition was determined by high-performance thin-layer chromatography.

#### 2.1.5. Cell Viability

Historical data were gathered over time from different experiments and the mean percentage of relative viability of the positive and the optical density (OD) of the negative control obtained in this study were compared to historical values and defined thresholds [17].

### 2.2. Skin Irritation Assay

#### 2.2.1. Test Substances

All solid and liquid test chemicals (Merck) were selected from the minimum list of reference chemicals for determination of reproducibility and predictive capacity of similar or modified RhE skin irritation test methods, as described in guidance document No. 220 [3] (Table 1). Additionally, PBS and 5% SDS were included in each assay as a negative and positive control, respectively.

All chemicals were evaluated in triplicate in three experimental replicates. Additionally, all chemicals were pre-checked prior to use for direct MTT reduction or color interference. When applicable, final relative viability values were corrected appropriately using freeze-killed and/or living-tissue controls according to the protocols described in the guidance document No. 220 [3].

#### 2.2.2. Electrochemical Impedance Spectroscopy (EIS) Analysis

**Figure 1:**
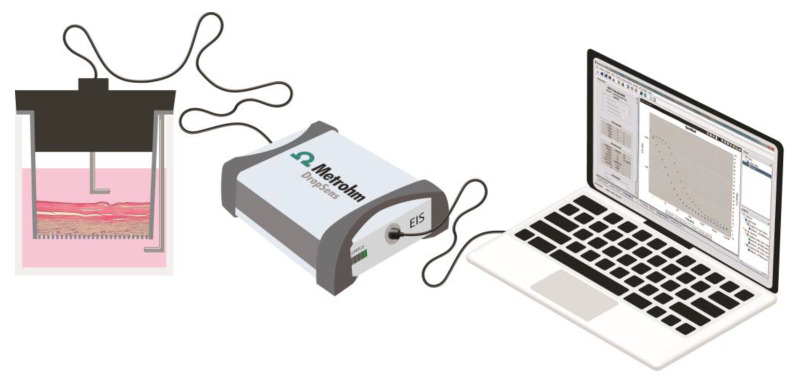
Experimental set-up for EIS analysis.

EIS evaluation was performed using an electrochemical impedance spectroscope µStat-I 400s (Metrohm Dropsens, Asturias, Spain). Impedance was measured at 25 logarithmically distributed points over a frequency range of [1 Hz–1 MHz] through a 10 mV sinusoidal AC pulse applied between a chopstick-like pair of Ag electrodes (one acting as working electrode and the other as pseudo-reference electrode) placed on the inside and outside of the insert at a fixed distance (Figure 1). Capacitance (C) was calculated as:

$$C=\frac{1}{2\pi fZ^{\prime \prime }}$$

where, *C* is the capacitance, f is the frequency, and *Z*″ is the imaginary term of impedance. Data were analyzed using DropView 8400 software (Metrohm Dropsens).

#### 2.2.3. Procedural Conditions

In order to assess skin irritation, an OECD TG 439 complying protocol was used [2]. Briefly, capacitance was assessed in each model before chemical application. Afterwards, QileX-RhE models were exposed to the reference chemicals by applying 50 µL (liquids) or 50 mg (solids) in triplicate. After 15 min of exposure at room temperature, test chemicals were decanted and the QileX-RhE models were washed abundantly in a gentle, continuous flow of PBS. After washing, the models were post-washed for 15 min in culture medium. At the end of the post-wash, each model was transferred to a new culture plate containing 1 mL of culture medium and the models were incubated for a total of 42 h at 37 °C. Capacitance was assessed at 2 h, 24 h, and 42 h of incubation and relative cell viability was assessed by MTT after 42 h of incubation.

#### 2.2.4. Relative Viability Evaluation

Relative cell viability was assessed by the 3-(4,5-dimethylthiazol-2-yl)-2,5-diphenyl bromide (MTT)-based reduction assay (Merck). In this assay, MTT, a yellowish tetrazolium salt, is reduced to a purple formazan salt by the primary action of mitochondrial succinate dehydrogenase in viable cells.

Briefly, once models were treated with the respective test chemical and having been washed and incubated for the relevant time, 0.5 mL of MTT dissolved in a mixture of DMEM and Ham’s F12 (Merck) was added 1:1 at a final concentration of 0.5 mg/mL over the insert and an additional 0.5 mL was added in the culture plate and incubated at 37 °C for 180 min. After the incubation time, the medium containing the MTT was removed and the formazan salts were solubilized by adding 1 mL of DMSO (0.5 mL on the insert and 0.5 mL on the culture plate) and incubating for 15 min at room temperature.

Finally, three 100 µL aliquots of the extracted solution of each model were transferred to a 96-well plate and the OD was assessed at 570 nm for 0.1 s in a VICTOR multilabel plate reader (PerkinElmer, MA, USA). According to OECD TG 439, a substance is classified as an irritant when the relative viability is less than (<) 50%.

#### 2.2.5. Reliability and Accuracy Assessment

Statistical analyses were performed using GraphPad Prism 9 software (GraphPad Software, San Diego, CA, USA).

In order to predict skin irritation, the capacitance (C) data obtained before and after chemical treatment were normalized for each evaluated frequency and time point as C_x_/C_0_, where C_0_ is the initial capacitance value obtained before chemical application and C_x_ is the capacitance value after 2, 24, or 42 h.

Optimal capacitance frequency was determined using a frequency-dependent spectrum. Frequency in which capacitance displayed a maximum response was selected for irritancy prediction.

The optimal evaluation time was determined by ROC curves and AUC analysis. Similarly, the best threshold for classification was selected based on the value that displayed the best accuracy for distinguishing irritants from non-irritants.

Finally, best classification obtained by capacitance analysis was compared with conventional classification using relative viability data obtained by the MTT assay.

#### 2.2.6. Acceptance Criteria

A run was considered qualified when the following criteria were met:

(1) Initial barrier integrity according to TEER was ≥600 and ≤2500 Ωcm^2^;
(2) MTT absorbance value of the negative control was ≥0.40 and ≤0.70;
(3) Cell viability of positive control was <50%;
(4) Cell viability variability between tissue replicates was SD ≤ 18.

In case TEER values were outside accepted ranges, QileX-RhE models were discarded for further testing. In case either negative or positive control values were outside accepted ranges, all tested chemicals included in the run were considered non-qualified and were repeated. In case the cell viability variability between tissue triplicates of a test chemical was >18, the test chemical was re-tested.

## 3. Results

### 3.1. Quality Control of Test Method Components

#### 3.1.1. Morphology

**Figure 2:**
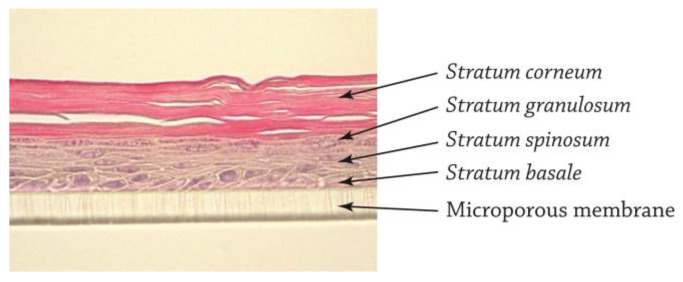
Histological evaluation of QileX-RhE model.

The QileX-RhE models were a pluristratified epithelium of between five to seven cell layers, reflecting a highly specialized pattern of differentiation consisting of stratum basale, stratum spinosum, stratum granulosum, and stratum corneum (Figure 2).

#### 3.1.2. Barrier Function

**Figure 3:**
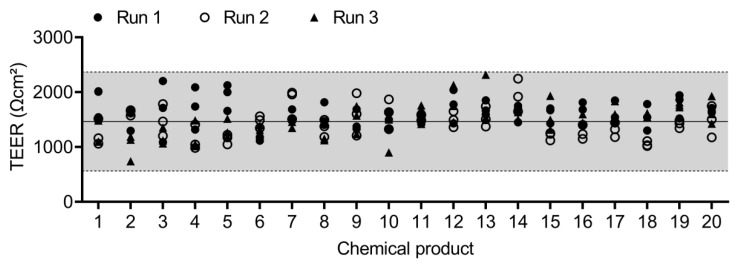
TEER values of QileX-RhE models used in this study.

The epithelial integrity of the QileX-RhE models used in this study is shown in Figure 3.

#### 3.1.3. Lipid Profile

The lipid profile revealed that the main epidermal lipid classes are present in the QileX-RhE model (Table 2), including phospholipids, cholesterol sulphate, ceramides, free fatty acids, cholesterol, triglycerides. and cholesterol esters.

#### 3.1.4. Cell Viability

**Figure 4:**
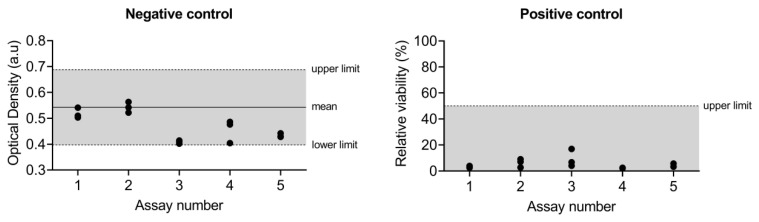
Negative and positive quality control data of the different assays included in this study.

Negative and positive control values of the assays included in this study are shown in Figure 4.

#### 3.1.5. Capacitance Evaluation

**Figure 5:**
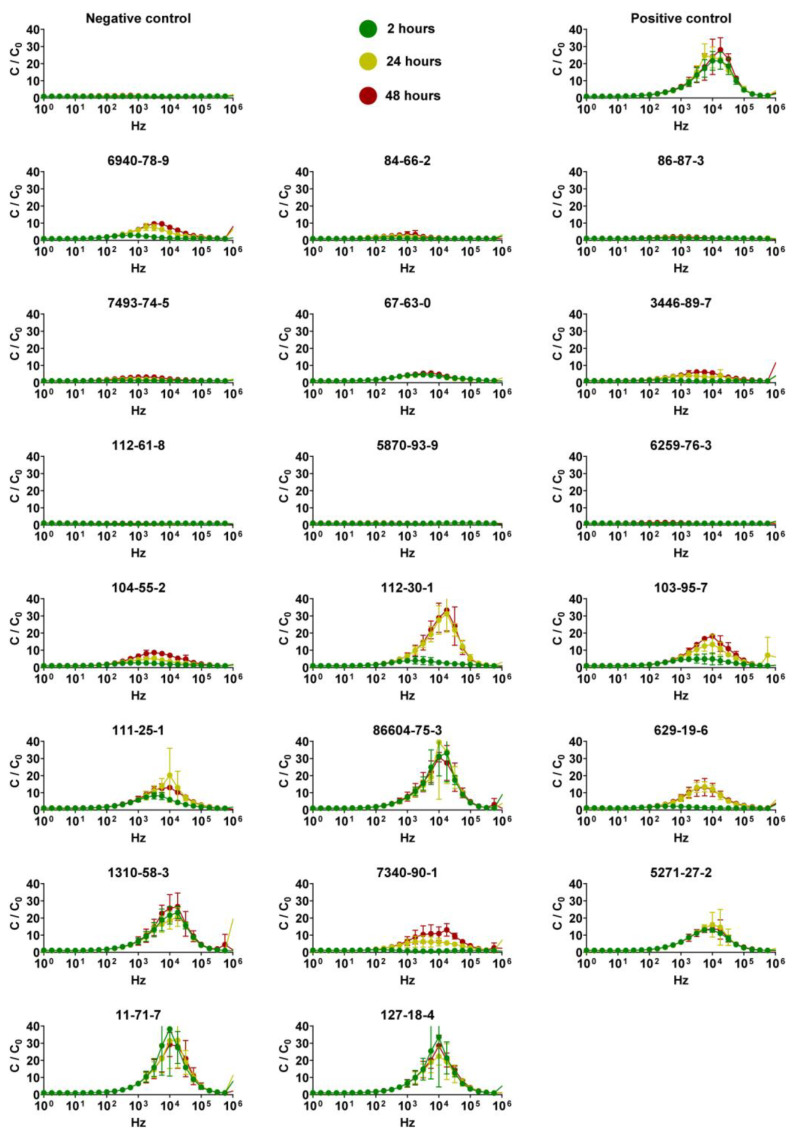
Determination of optimal frequency using a frequency dependent spectrum. Data are presented as normalized capacitance (*y*-axis) versus frequency (*x*-axis) measured at 2, 24, and 42 h. Data are shown as mean ± SD; n = 3.

Capacitance analysis of QileX-RhE models was carried out to evaluate variations before and after test substance application at three different time points (2, 24, and 42 h) over a frequency range of [1 Hz–1 MHz] (Figure 5).

As presented in Figure 5, the capacitance spectrum showed a maximum response after the application of the positive control, as in some of the test chemicals, when evaluated at 17 kHz; therefore, 17 kHz was selected as the frequency to evaluate the effects of the different test chemicals on the QileX-RhE model (Table 3).

**Figure 6:**
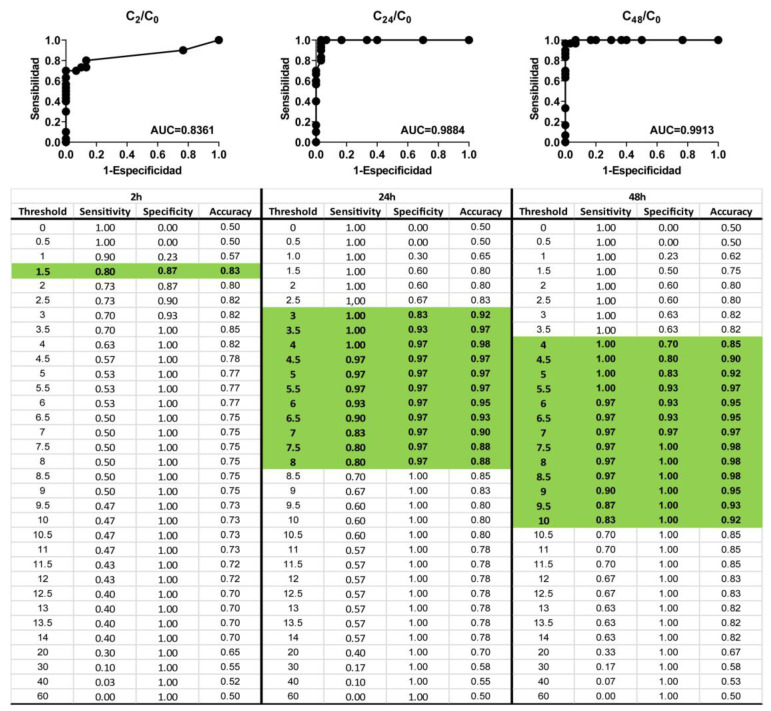
ROC curve analysis for 20 reference chemicals. Highlighted fields represent the cut-off values complying with OECD TG 439 requirements.

Subsequently, in order to determine the evaluation time that resulted in the best predictive performance, a ROC curve analysis was used. The ROC curve analysis was conducted with normalized capacitance data at 17 kHz for the 20 reference chemicals evaluated after 2, 24, and 42 h post-exposure. As presented in Figure 6, normalized capacitance analysis has shown a predictive performance that complied with the OECD TG 439 requirements in all cases (sensitivity ≥ 80%; specificity ≥ 70%; accuracy ≥ 75%). Overall, the optimal cut-off values were determined to be: 1.5 for normalized capacitance measured 2 h post-exposure, achieving an AUC of 0.8361; 4 for normalized capacitance measured 24 h post-exposure, achieving an AUC of 0.9884; and a range between 7.5 and 8.5 for normalized capacitance measured 42 h post-exposure, achieving an AUC of 0.9913.

In order to evaluate the within-laboratory reproducibility of the prediction method based on normalized capacitance, the data obtained after 42 h post-exposure using an irritant threshold of 7.5 were selected as suggested by the ROC analysis (Table 4). Overall decisions were consistent except for Di-n-propyl disulphide (CASRN: 629-19-6), which was incorrectly predicted once. Therefore, we obtained 95% (19/20) concordance in the decision of “non-irritant” or “irritant”, complying with the criteria of within-laboratory reproducibility ≥ 90% defined in OECD TG 439.

**Figure 7:**
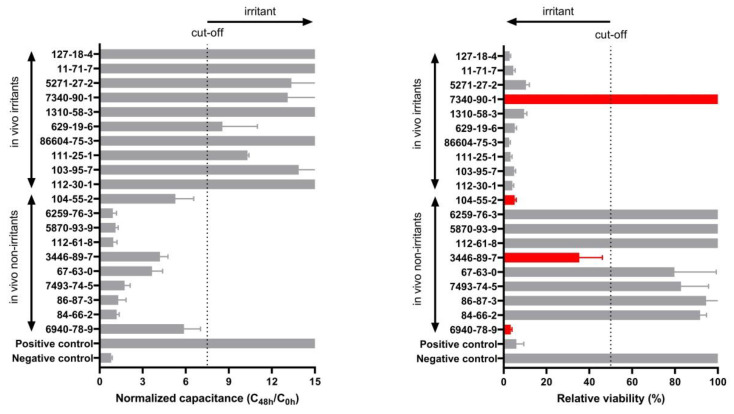
QileX-RhE normalized capacitance after 42 h at 17 kHz versus relative viability after 42 h. Red color indicates falsely predicted substances. Grey color represent correctly predicted substances. Dotted line indicates cut-off (7.5 in normalized capacitance and 50% in relative viability).

Finally, we compared the results with the classification obtained using the MTT viability assay as it is the test method of choice suggested in the OECD TG 439. As shown in Figure 7, predictions obtained using capacitance achieved 100% sensitivity, specificity, and accuracy, while using the MTT relative viability assay on the same samples a 90% sensitivity, 70% specificity, and 80% accuracy was obtained.

## 4. Discussion

In response to the European priority to replace and minimize animal testing, different RhE models have been developed as an alternative method for the assessment of skin irritation, some of which have been validated and included in OECD TG 431 [2]. In this study, we make use of the QileX-RhE model (previously referred to as DIY-RhE), a reconstructed human epidermis model developed by our own laboratory to assess skin corrosion [17]. Histologically, QileX-RhE reflects the ultrastructure of a native epidermis, revealing a keratinized pluristratified squamous epithelium structured in *stratum basale*, *spinosum*, *granulosum,* and *corneum*. The *stratum corneum* is the uppermost layer of the epidermis, which is composed of protein-enriched corneocytes embedded in a lipid-enriched intercellular matrix [19]. For the correct formation of the stratum corneum, the presence of the main lipid classes (cholesterol, ceramides, and free fatty acids) is indispensable. Analysis of the lipid content of the stratum corneum shows that the main lipid classes are present in the QileX-RhE model, although in different proportions than in the human epidermis. Recent studies show that, in a healthy stratum corneum, cholesterol accounts for 27% of the total lipid composition, ceramides for 50%, and free fatty acids for 10–15% [20,21]; however, these results differ from those obtained by Ponec et al. in their 2002 study analyzing normal human epidermis [18], which have been used as a comparison in this study. These differences in the lipid composition of the stratum corneum can be explained by the fact that the lipid composition varies depending on the anatomical region analyzed [22]. The results obtained after analysis of the QileX-RhE model shows a cholesterol content of 28%, a ceramide content of 26%, and a free fatty acid content of 6%. Based on these results, the ceramide content is significantly lower than observed in healthy stratum corneum (50%) but very similar to the SkinEthic RHE model (26.5%) and higher than the EpiDerm^®^ model (18.5%). Ceramides are the main lipid component of the lamellae found in the intercellular spaces of the stratum corneum and are responsible for providing the barrier effect in the epidermis [23]. Thus, based on the ceramide content, it is possible that the barrier effect of the QileX-RhE model is lower than the normal human skin; however, it would be equal to or higher than the validated RhE models. This is in line with the results obtained in the evaluation of the barrier effect by TEER measurement, where TEER values of QileX-RhE models are higher than EpiDerm^®^ but lower than SkinEthic RHE [24]; therefore, it is expected that the performance of the QileX-RhE model in the assessment of skin irritation is in line with the results obtained by the validated reference methods, as the models are mechanistically similar.

For over a decade, the evaluation of cell viability by enzymatic conversion of MTT and the identification of irritants by the decrease in cell viability below the defined thresholds has been the way to go. Although it has been a major breakthrough for animal welfare and the standardization and worldwide implementation of alternative methods, relying on MTT viability tests presents several flaws that highly limit the multiplexing of assays in order to achieve a broader scope of safety assessment. On one side, test chemicals may directly interfere with cell viability measurements by non-enzymatically reducing MTT or by absorbing light in the same range as formazan [25,26]. On the other side, although MTT usually correlates with the number of viable cells, the rate of MTT reduction reflects the metabolic activity of cells (the rate of glycolytic NADH production), which may change after chemical exposure, leading to results that may not relate to the number of viable cells [27]. Last but not least, the formazan crystals need to be solubilized in organic solvents, which destroys cell architecture and limits the ability to multiplex with complementary techniques, unless other assays precede the MTT assay.

In this study, we evaluate the usefulness of a technique based on a non-destructive and non-cell-interfering EIS analysis as a predictive method for skin irritation, following the standardized criteria described in OECD TG 439 and guidance document No. 220 for the development of new RhE-based predictive methods [2,3]. The results obtained in this study show that it has been possible to integrate EIS assessment into the standardized protocols for the assessment of skin irritation in RhE models without interfering with the assessment of relative cell viability by MTT. Thus, through the study of capacitance, it has been possible to study the evolution of cell damages after the application of chemical compounds after 2 h, 24 h, and 42 h. In the conventional methodology using the MTT assay, viability measurements must be performed after a sufficient post-treatment incubation period to allow for recovery from weak cytotoxic effects and for the appearance of clear cytotoxic effects [2]. However, no distinction is made between those chemicals since MTT assay can only be performed once per tissue model, and thus, is usually evaluated 42 h post-treatment leading to a final irritancy prediction. In contrast, EIS analysis allows the comparison of individual pre- and post-treatment capacitance values at different times, which allows the evaluation of strong or reversible effects on the epidermis models. In our results, no reversible effects have been identified for the selected test chemicals; however, irritant responses identified at 24 or 42 h post-exposure were not initially detected after 2 h post-exposure in some cases (chemicals CASRN 629-19-6 and CASRN 7340-90-1), indicating different grades of irritancy that could be subclassified in order to refine irritancy assessment. 

In order to assess which frequency offered the best predictive performance, we evaluated capacitance on a broad frequency rang [1Hz–1MHz]. In the case of QileX-RhE, we observed a peak capacitance response after chemical application at 17 kHz, and thus data at 17 kHz were used for further analysis. Subsequently, ROC analysis was performed to evaluate predictive performance at the different evaluated times. We observed a prediction that complied with OECD TG 439 requirements at each evaluated time (2, 24, and 42 h), although assuming different sensitivity, specificity, and accuracy. As suggested, this could be attributed to the different irritancy potential of each chemical that is usually overlooked using the MTT viability assay, which only evaluates irritancy after 42 h. In any case, AUC was estimated to be 0.8361 at 2 h, 0.9884 at 24 h, and 0.9913 at 42 h, suggesting an exceptional discriminative validity of the test method [28] in comparison to what can be achieved with MTT on the validated methods [29]. As a final comparison, we selected the best-performing analysis and compared the results with those of our own using the MTT assay. In this case, we obtained 100% sensitivity, 100% specificity, and 100% accuracy with 95% within-laboratory reproducibility using the capacitance analysis in contrast to 90% sensitivity, 70% specificity, 80% accuracy, and 100% within-laboratory reproducibility achieved with the MTT assay. This last result is in line with the prediction obtained on the validation studies of the RhE reference methods using the same test chemicals [24,29,30,31] and supports the use of QileX-RhE as a new structurally and mechanistically similar RhE test method for skin irritancy testing.

The difference in classification between capacitance and cell viability data could be explained as skin irritation being the result of a skin barrier disruption, the activation of innate immune responses, and the dilation and increased permeability in the endothelial cells of the blood vessels [32]. As RhE-based test methods lack any vasculature, the MTT assay bases its prediction on cell death as an initiating event of an inflammatory cascade [2]. However, cell death may not result in an inflammatory cascade in all cases, as different pathways leading to cell death play a fundamental role in the release of pro-inflammatory cytokines [33]. For example, a test chemical inducing cell death via apoptosis will not trigger an inflammatory cascade that leads to in vivo irritation; however, it will be falsely predicted as an irritant according to the MTT assay. In contrast, previous studies have suggested that capacitance ratios are higher in necrosis than in apoptosis [34], making possible the differentiation of whether a test chemical will trigger an inflammatory cascade or not. Consequently, and as suggested by our results, capacitance analysis seems to be more related to the initiating events that lead to the inflammatory cascade that causes in vivo skin irritation and represents a valuable method to evaluate skin irritation on in vitro RhE models. In order to overcome this limitation, MTT-based studies are often complemented with cytokine release assays to complement the viability data. However, an unacceptable 55% sensitivity to detect irritants through the analysis of IL-1α was obtained on the EPISKIN prediction model [35], while no direct inverse correlations were found between the MTT values of skin sensitizers and the amount of IL-8 or IL-1α in the SkinEthic model [36].

It is important to note that, to date, only the MTT assay is expected to be used within test protocols for the evaluation of skin irritancy on RhE. However, in the interest of refinement of alternative methods and the promotion of their use in broader fields, regulators must acknowledge different scientifically sound methodologies that demonstrate a comparable performance and offer novel perspectives on cell processes that could be translated to human health safety assessments.

## 5. Conclusions

In conclusion, this study demonstrates the utility of using electrochemical impedance spectroscopy as a stand-alone tool to evaluate skin irritation in reconstructed human epidermis models. The results showed that EIS can differentiate between irritant and non-irritant test chemicals and can be used as a single method for the evaluation of skin irritation in RhE models, with a significant increase in the capacitance values observed in the irritant samples. These findings suggest that EIS could potentially replace the use of animal testing in the evaluation of the irritation potential of chemicals and could be a valuable addition to the in vitro testing strategies outlined in the OECD Test Guideline 439. Further research is needed to validate the use of EIS in other RhE models and to fully understand its capabilities as a tool for evaluating skin irritation.

## Figures and Tables

**Table 1:** List of test chemicals used in this study.

N°	Chemical Product	CASRN	In Vivo GHS	Physical State
1	1-Bromo-4-chlorobutane	6940-78-9	NC	Liquid
2	Diethyl phthalate	84-66-2	NC	Liquid
3	Naphthalene acetic acid	86-87-3	NC	Solid
4	Allylphenoxy-acetate	7493-74-5	NC	Liquid
5	Isopropanol	67-63-0	NC	Liquid
6	4-Methyl-thio-benzaldehyde	3446-89-7	NC	Liquid
7	Methyl stearate	112-61-8	NC	Solid
8	Heptyl butyrate	5870-93-9	NC	Liquid
9	Heptyl salicylate	6259-76-3	NC	Liquid
10	Cinnamaldehyde	104-55-2	NC	Liquid
11	1-Decanol	112-30-1	CAT 2	Liquid
12	Cyclamen aldehyde	103-95-7	CAT 2	Liquid
13	1-Bromohexane	111-25-1	CAT 2	Liquid
14	2-Chloromethyl-3,5-dimethyl-4-methoxypyridine HCl	86604-75-3	CAT 2	Solid
15	Di-n-propyl disulphide	629-19-6	CAT 2	Liquid
16	Potassium hydroxide (5% aq.)	1310-58-3	CAT 2	Liquid
17	Benzenethiol, 5-(1,1-dimethylethyl)-2-methyl	7340-90-1	CAT 2	Liquid
18	1-Methyl-3-phenyl-1-piperazine	5271-27-2	CAT 2	Solid
19	Heptanal	111-71-7	CAT 2	Liquid
20	Tetrachloroethylene	127-18-4	CAT 2	Liquid

**Table 2:** Lipid composition of QileX-RhE, validated reference method and native epidermis.

Lipids	QileX-RhE	SkinEthic RHE *	EpiDerm ^®,^*	Epidermis *
Phospholipids	10.7 ± 1.8	17.0 ± 10.5	36.5 ± 2.7	36.5 ± 4.1
Sphingomyelin	1.7 ± 0.3	2.8 ± 1.3	8.2 ± 1.5	8.9 ± 1.6
Phosphatidylcholine	3.0 ± 0.6	6.4 ± 3.8	13.6 ± 2.4	11.2 ± 0.8
Phosphatidylserine	0.6 ± 0.1	1.1 ± 0.7	3.2 ± 0.7	3.9 ± 0.3
Phosphatidylinositol	0.6 ± 0.2	1.8 ± 1.2	4.3 ± 0.8	2.2 ± 0.8
Phosphatidylethanolamine	4.1 ± 0.8	4.9 ± 4.0	7.1 ± 1.6	10.3 ± 0.8
Cholesterol sulfate	3.7 ± 0.4	3.8 ± 2.0	5.8 ± 1.2	5.0 ± 1.6
Ceramides	26.0 ± 0.6	26.5 ± 12.2	18.5 ± 3.5	12.1 ± 1.8
Free fatty acids	5.9 ± 1.7	6.9 ± 3.9	2.6 ± 0.5	7.8 ± 1.2
Cholesterol	28.1 ± 0.7	19.5 ± 9.5	14.8 ± 1.3	17.7 ± 3.2
Di-triglycerides	16.2 ± 1.6	12.6 ± 8.6	10.5 ± 2.2	8.9 ± 3.7
Cholesterol esters	6.0 ± 1.8	6.5 ± 4.4	2.7 ± 1.1	7.0 ± 0.4

* According to Ponet et al. 2002 [18].

**Table 3:** Normalized capacitance data at 17 kHz after chemical exposure.

	Normalized Capacitance [17 kHz]		
Test Chemical	2 h	24 h	42 h
**Negative control**	0.96 ± 0.02	0.87 ± 0.10	0.81 ± 0.07
**Positive control**	21.66 ± 4.94	22.50 ± 1.24	28.04 ± 7.04
**In vivo non-irritants (GHS No Category)**			
6940-78-9	1.17 ± 0.22	3.17 ± 0.48	5.88 ± 1.15
84-66-2	0.97 ± 0.15	1.07 ± 0.10	1.19 ± 0.17
86-87-3	1.26 ± 0.09	1.24 ± 0.23	1.30 ± 0.34
7493-74-5	1.08 ± 0.14	1.17 ± 0.23	1.74 ± 0.38
67-63-0	3.13 ± 0.22	2.89 ± 0.20	3.65 ± 0.74
3446-89-7	1.05 ± 0.06	4.10 ± 3.49	4.20 ± 0.55
112-61-8	1.04 ± 0.08	0.88 ± 0.09	0.95 ± 0.27
5870-93-9	1.17 ± 0.14	1.07 ± 0.18	1.11 ± 0.18
6259-76-3	1.00 ± 0.15	0.96 ± 0.13	0.92 ± 0.25
104-55-2	1.74 ± 0.65	2.83 ± 0.27	5.28 ± 1.28
**In vivo irritants (GHS Category 2)**			
112-30-1	2.55 ± 1.00	31.22 ± 10.71	33.29 ± 12.00
103-95-7	4.01 ± 2.27	10.94 ± 3.70	13.88 ± 4.53
111-25-1	4.41 ± 2.27	13.01 ± 9.56	10.30 ± 0.10
86604-75-3	33.16 ± 16.72	34.00 ± 18.61	27.41 ± 10.15
629-19-6	1.12 ± 0.35	8.31 ± 2.24	8.56 ± 2.44
1310-58-3	23.17 ± 3.50	20.58 ± 5.44	26.61 ± 7.99
7340-90-1	0.89 ± 0.17	5.45 ± 1.19	13.10 ± 3.63
5271-27-2	11.10 ± 1.54	14.60 ± 10.37	13.36 ± 5.62
111-71-7	27.52 ± 9.31	31.72 ± 15.98	28.20 ± 12.85
127-18-4	21.42 ± 9.29	19.26 ± 10.44	20.21 ± 4.03

**Table 4:** Within-laboratory reproducibility of the QileX-RhE normalized capacitance. Data are presented as mean ± SD.

	Normalized Capacitance (42 h) [17 kHz]			
Test Chemical	Run 1	Run 2	Run 3	Mean
**Negative control**	0.88 ± 1.13	0.74 ± 0.04	0.8 ± 0.32	0.81 ± 0.07
**Positive control**	36.08 ± 9.19	25.07 ± 2.55	22.97 ± 13.73	28.04 ± 7.04
**In vivo non-irritants (GHS No Category)**				
6940-78-9	5.34 ± 0.79	5.11 ± 0.10	7.2 ± 2.39	5.88 ± 1.15
84-66-2	1.00 ± 0.14	1.34 ± 0.42	1.23 ± 0.29	1.19 ± 0.17
86-87-3	0.85 ± 0.08	1.90 ± 0.28	1.14 ± 0.09	1.30 ± 0.34
7493-74-5	1.31 ± 0.23	1.92 ± 0.50	2.00 ± 0.84	1.74 ± 0.38
67-63-0	2.87 ± 0.93	3.75 ± 0.98	4.33 ± 1.33	3.65 ± 0.74
3446-89-7	3.59 ± 1.78	4.67 ± 2.62	4.35 ± 2.24	4.20 ± 0.55
112-61-8	0.95 ± 0.17	1.21 ± 0.12	0.68 ± 0.05	0.95 ± 0.27
5870-93-9	1.13 ± 0.11	1.28 ± 0.03	0.92 ± 0.04	1.11 ± 0.18
6259-76-3	0.97 ± 0.05	1.14 ± 0.14	0.65 ± 0.09	0.92 ± 0.25
104-55-2	4.02 ± 0.27	5.24 ± 0.42	6.57 ± 1.28	5.28 ± 1.28
**In vivo irritants (GHS Category 2)**				
112-30-1	36.66 ± 8.45	41.67 ± 2.66	19.55 ± 10.43	33.29 ± 12.00
103-95-7	15.69 ± 6.58	17.22 ± 2.91	8.73 ± 4.88	13.88 ± 4.53
111-25-1	10.25 ± 0.49	10.23 ± 0.89	10.42 ± 3.41	10.30 ± 0.10
86604-75-3	36.55 ± 10.60	29.21 ± 5.99	16.48 ± 8.15	27.41 ± 10.15
629-19-6	9.50 ± 0.70	10.38 ± 1.03	5.79 ± 2.30	8.56 ± 2.44
1310-58-3	20.26 ± 4.76	35.58 ± 14.10	23.98 ± 8.76	26.61 ± 7.99
7340-90-1	9.63 ± 0.69	12.8 ± 0.78	16.88 ± 16.24	13.10 ± 3.63
5271-27-2	8.81 ± 2.09	11.64 ± 4.63	19.64 ± 13.67	13.36 ± 5.62
111-71-7	25.15 ± 5.44	42.30 ± 4.93	17.16 ± 2.99	28.20 ± 12.85
127-18-4	19.89 ± 3.37	24.39 ± 4.54	16.35 ± 3.21	20.21 ± 4.03

## Data Availability

All the obtained data used to support the findings of this study are available from the corresponding author upon reasonable request.
